# The genus *Hygrocrates* Deeleman-Reinhold, 1988 (Araneae, Dysderidae) in Turkey

**DOI:** 10.3897/zookeys.85.927

**Published:** 2011-03-07

**Authors:** Kadir Boğaç Kunt, Ersen Aydın Yağmur, Recep Sulhi Özkütük, Rahşen S. Kaya

**Affiliations:** 1 Eserköy Sitesi 9/A Blok No:7 TR-06530 Ümitköy, Ankara, Turkey; 2 Ege University, Science Faculty, Biology Department, Zoology Section, TR-35100 İzmir, Turkey; 3 Department of Biology, Faculty of Science, Anadolu University, TR- 26470 Eskişehir, Turkey; 4 Department of Biology, Faculty of Arts and Sciences, Uludağ University, TR-16059, Bursa, Turkey

**Keywords:** Dysderidae, *Hygrocrates*, new species, systematic key, Turkey

## Abstract

A new species, *Hygrocrates deelemanus* Kunt & Yağmur **sp. n.**, is described on the basis of both sexes from the Mediterranean region of Turkey. Detailed morphological descriptions, diagnosis and figures of the copulatory organs of both Turkish species are presented. An identification key is presented for all the currently known species of *Hygrocrates*.

## Introduction

Hygrocrates Deeleman-Reinhold, 1988 is a small dysderid genus which contains only three previously described species: Hygrocrates caucasicus Dunin, 1992; Hygrocrates georgicus (Mcheidze, 1972) and Hygrocrates lycaoniae (Brignoli, 1978). Hygrocrates caucasicus and Hygrocrates georgicus are endemic to Georgia**,** while Hygrocrates lycaoniae is known from Rhodes and Turkey*. Hygrocrates lycaoniae* was originally ascribed to Harpactocrates Simon, 1914. In their revision of Dysderinae spiders of the Mediterranean region, Deeleman-Reinhold and Deeleman (1988) transferred Hygrocrates lycaoniae to the new genus Hygrocrates, which was differentiated from Harpactocrates as follows: posterior median eyes closer to each other; anterior cheliceral teeth smaller in the basal region, and the presence of a subapical apophysis on the male palp. Hygrocrates georgicus, originally ascribed to Harpactocrates, was only provisionally transferred to the genus Hygrocrates by Deeleman-Reinhold and Deeleman (1988); see also Dunin (1992) who reported that the female holotype of this species was lost. Subsequently, the third, new species Hygrocrates caucasicus was described by Dunin (1992), based on two male specimens from the Georgia.

To date, only Hygrocrates lycaoniae has been recorded from Turkey based on only one male in a sample collected from outside Körükini Cave (Konya province, Beyşehir District, Çamlık town). After the original description by Brignoli (1978), Deeleman-Reinhold and Deeleman (1988) redescribed this species based on male and female specimens collected from Greece. During our surveys of the Turkish spider fauna we collected some specimens of Hygrocrates lycaoniae, in addition to some specimens that were impossible to place in any of the known species of Hygrocrates. In this paper, a new species of the genus, namely Hygrocrates deelemanus sp. n., is described based on both sexes, collected from the southern region of Turkey. The characteristic features of both species, including photographs of the prosoma and of the copulatory organs, are provided for comparative purposes.

## Material and methods

**Figure 1.**
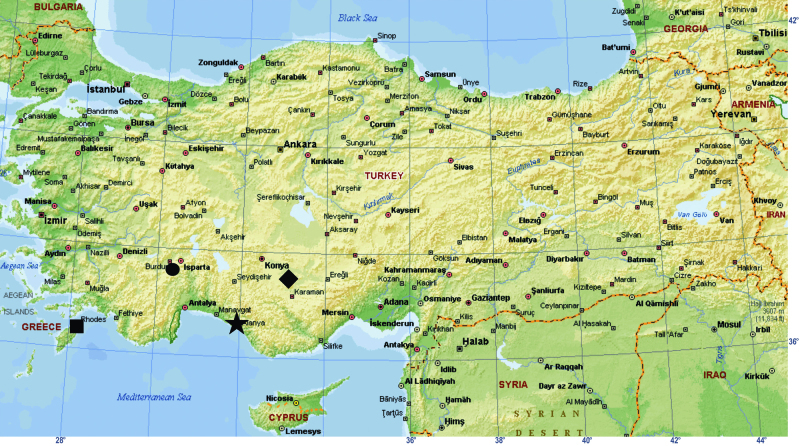
Map showing the localities from which Hygrocrates specimens have been recorded. ★ the type locality of Hygrocrates deelemanus sp. n.; ♦ the type locality of Hygrocrates lycaoniae; ⚫ locality of Hygrocrates lycaoniae from Turkey; ■ locality of Hygrocrates lycaoniae from Rhodos island (Greece).

All specimens were collected from two different localities in Turkey (Fig. 1). The specimens were collected by sifting of leaf litter and preserved in 70% ethanol. Digital images of the pedipalp and vulva were taken with a Leica DFC295 digital camera attached to a Leica S8AP0 stereomicroscope and 5–15 photographs were taken in different focal planes and combined. Photographic images were edited using Photoshop CS2 and Corel-DRAW X3 was used to create the plates. All measurements are in mm. Terminology for the body measurements and copulatory organ structures follows Deeleman-Reinhold and Deeleman (1988) and Chatzaki and Arnedo (2006). The following terminology is used for the male palp: apical apophysis of the male palp, **Embolus**; subapical apophysis of the male palp, **Apophysisa**; and posterior apophysis of the male palp, **Apophysisb**.

Material treated herein is deposited in the personal collection of Kadir Boğaç Kunt (**cKBK**, Ankara, Turkey) and in the Senckenberg Museum (**SMF**, Frankfurt am Main, Germany). The following abbreviations are used in the text: **AL**, abdominal length; **CL**, carapace length; **CWmax**, maximum carapace width; **CWmin**, minimum carapace width; **AME**, anterior median eyes; **PLE**, posterior lateral eyes; **PME**, posterior median eyes; **AMEd**, diameter of anterior median eyes; **PLEd**, diameter of posterior lateral eyes; **PMEd**, diameter of posterior median eyes; **ChF**, length of cheliceral fang; **ChG**, length of cheliceral groove; **ChL**, total length of chelicera (lateral external view); **Ta**, tarsus; **Me**, metatarsus, **Ti**, tibia; **Pa**, patella; **Fe**, femur; **Tr**, trochanter; **C**, coxa; **D**, dorsal; **Pl**, prolateral; **Rl**, retrolateral; **V**, ventral.

## Key to the Hygrocrates species

**Table:** 

1	Male	2
–	Female	4
2	Bulbus straight, cylindrical; embolus lobe-shaped; apophysisa and apophysisb nearly same size	Hygrocrates caucasicus
–	Bulbus pyriform; embolus hook-shaped; apophysisa and apophysisb smaller than embolus	3
3	Transition between bulbus and distal continuation is gradual (Figs 30–33, 36)	Hygrocrates lycaoniae
–	Transition between bulbus and distal continuation is abrupt, clearly curved over 90° (Figs 16–19, 35)	Hygrocrates deelemanus sp. n.
4	Distalmost part of spermathecae linear	Hygrocrates georgicus
–	Distalmost part of spermathecae oval	5
5	Proximalmost part of spermathecae oval (Fig. 20)	Hygrocrates deelemanus sp. n.
–	Proximalmost part of spermathecae circular (Fig. 34)	Hygrocrates lycaoniae

## Taxonomy

**Hygrocrates Deeleman-Reinhold, 1988**

*H*. Deeleman-Reinhold, in Deeleman-Reinhold and Deeleman, 1988: 240, type Harpactocrates lycaoniae Brignoli, 1978.

### Hygrocrates deelemanus

Kunt & Yağmur sp. n.

urn:lsid:zoobank.org:act:3EBEFEC5-1A65-480F-BAB1-954463DCFC5C

**Figure 2.**
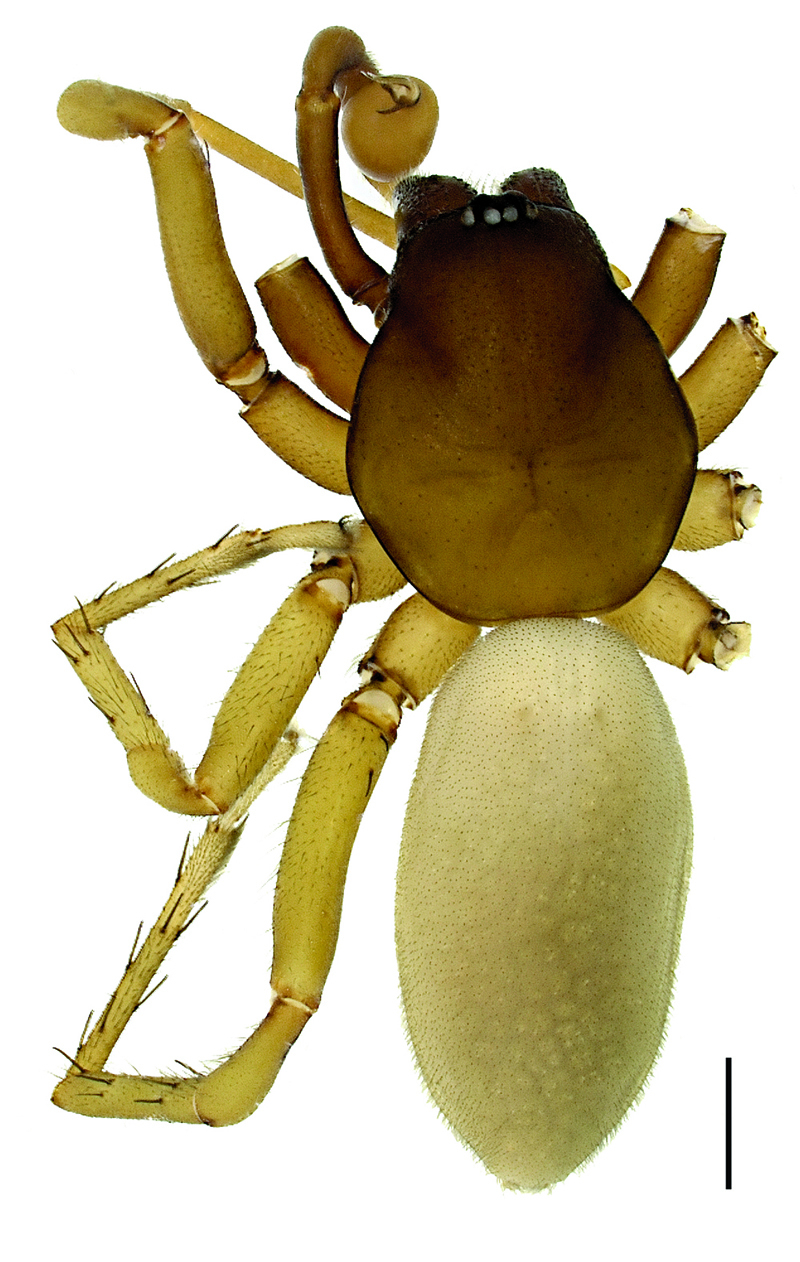
Hygrocrates deelemanus sp. n. (Holotype ♂); scale line: 1 mm.

Figs 2 [Fig F3] [Fig F4] [Fig F5] [Fig F6] 21 35 37

#### Material Examined:

Holotype ♂ (SMF) **Antalya Province**, Alanya District, Taşatan Plateau [36°38'33.20"N, 32°4'44.40"E], 09.I.2010, leg. K.B.Kunt. Paratypes: 1♀ (abdomen heavily damaged during dissection) (SMF), 1♂ (cKBK) same data as holotype.

#### Diagnosis:

The male palp of Hygrocrates deelemanus sp. n. is most similar to that of Hygrocrates lycaoniae. Bulbal apophyses are shorter than the embolus in both species. However, the transition between the bulbus and distal continuation is more abrupt in Hygrocrates deelemanus sp. n., clearly curved over 90°, whereas it is more gradual in Hygrocrates lycaoniae. In the vulva, the proximalmost part of the spermathecae is larger and wider than in Hygrocrates lycaoniae.

**Figures 3–10. F3:**
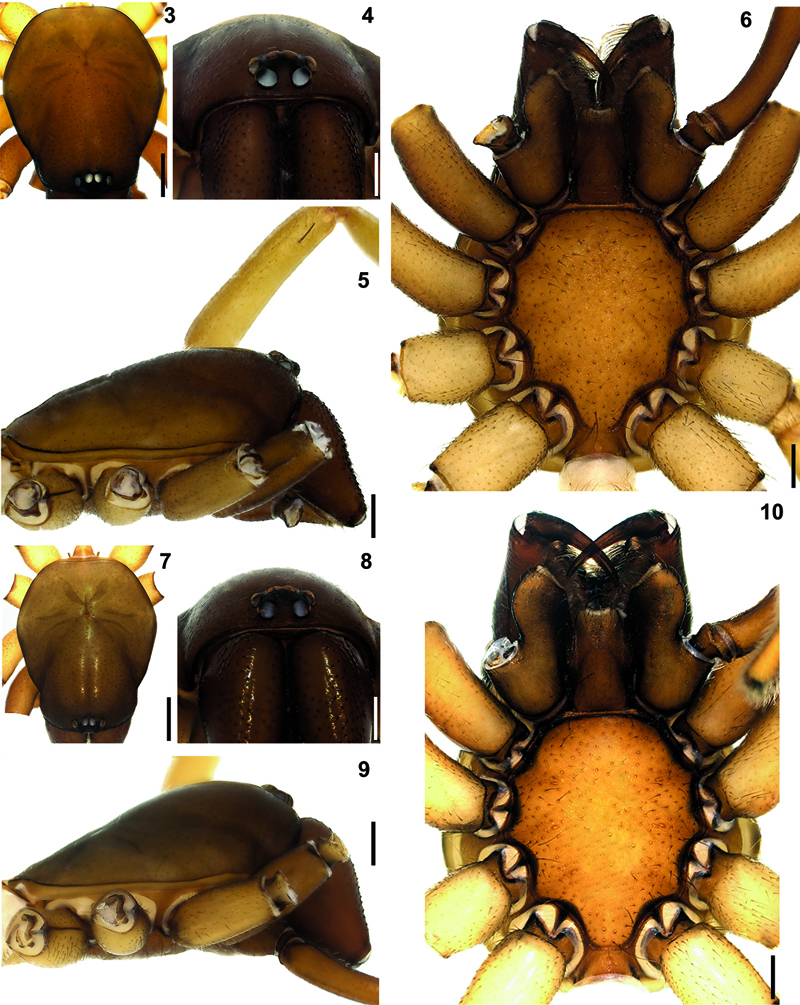
Hygrocrates deelemanus sp. n. **3** (♂), **7** (♀) carapace, dorsal view **4** (♂), **8** (♀) ditto, anterior view **5** (♂), **9** (♀) ditto, lateral view **6** (♂)**, 10** (♀) ditto, ventral view. Scale lines: (3, 5, 6, 7, 9, 10) 0.5 mm; (4, 8) 0.25 mm.

#### Derivatio nominis:

The specific name is given in honour of Dr. Christa L. Deeleman-Reinhold, a prominent Netherlander arachnologist who described the genus Hygrocrates.

#### Measurements (Holotype ♂ / Paratype ♀):

AL 3.84 / ?; CL 2.76 / 3.04; CWmax 2.28 / 2.40; CWmin 1.40 / 1.76; AMEd 0.16 / 0.19; PLEd 0.12 / 0.14; PMEd 0.10 / 0.11; ChF 0.77 / 0.82; ChG 0.46 / 0.52; ChL 1.20 / 1.50. Leg measurements are given in Table 1.

#### Description:

Carapace hexagonal-shaped, reddish brown, surface of carapace with small dark shallow depressions. Cephalic region dark brownish, narrow and clearly higher than thoracic region (Figs 2, 3, 5, 7, 9). Chilum triangular-shaped, distinct, coloured as carapace and chelicerae and broader in females (Figs 4, 8). Fovea short, straight and longitudinal (Figs 2, 3, 7). AME, PLE and PME closely grouped. Distance of AME-PLE shorter than PLE-PME. AME separated (Figs 4, 8). Labium, gnathocoxae and chelicerae brown. Labium and gnathocoxae covered with dark hairs; more densely so on gnathocoxae. Labium wider at the base. Gnathocoxae rounded laterally, with inwardly notched tips and blackish along the margins. Sternal border of gnathocoxae pentagonal-shaped, cheliceral region arrow-shaped with blunt tip (Figs 6, 10).

**Figure 11. F4:**
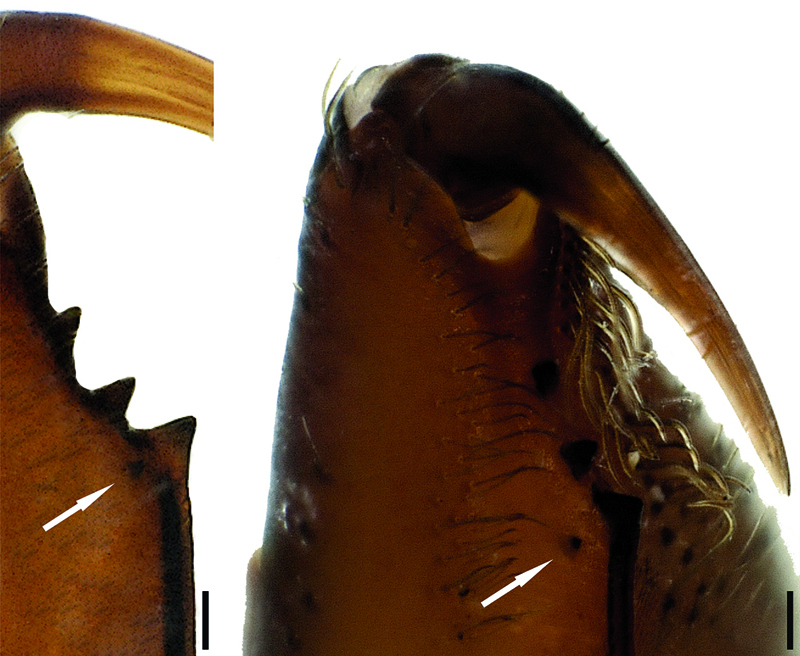
Cheliceral teeth of Hygrocrates deelemanus sp. n.

**Figures 12–15. F5:**
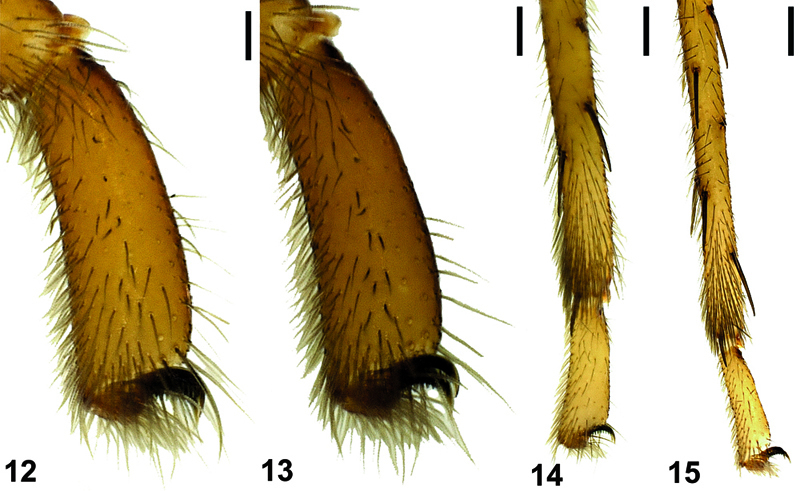
Tarsal and metatarsal scopulae of Hygrocrates deelemanus sp. n. (♂), **12** leg I, **13** leg II, **14** leg III, **15** leg IV. Scale lines: 0.25 mm

Chelicerae brownish with darker tubercules; basally broader in females and laterally swollen (Figs 4, 8). Cheliceral groove with four teeth: retromargin with four teeth, including one small and one large tooth at the base of the groove (Fig. 11). Sternum and abdomen yellowish brown, with thin blackish hairs over the entire surfaces. Legs yellowish brown. Palps and legs I and II darker than legs III and IV. Leg length formula: Leg I > Leg IV > Leg II > Leg III. Tarsi with two claws and claw tufts. All tarsi with fine tarsal scopulae. Legs III and IV with metatarsal scopulae (Figs 12–15). Coxae without spines. Details of leg spination are given in Table 2.

**Figures 16–19. F6:**
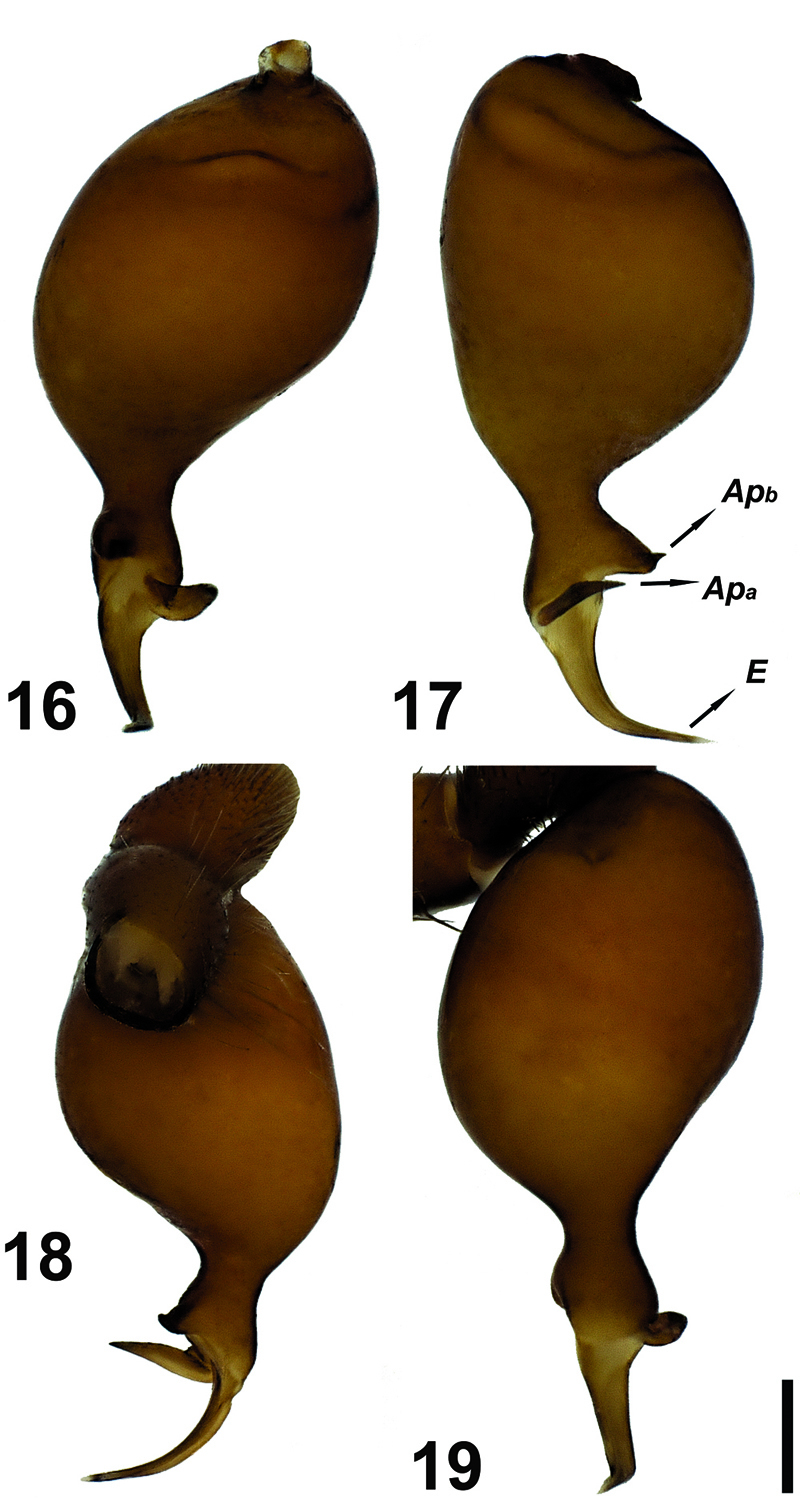
Male palp of Hygrocrates deelemanus sp. n. Abbreviations: Apa apophysisa Apb apophysisb E embolus. Scale line: 0.1 mm.

**Figures 20–21.**
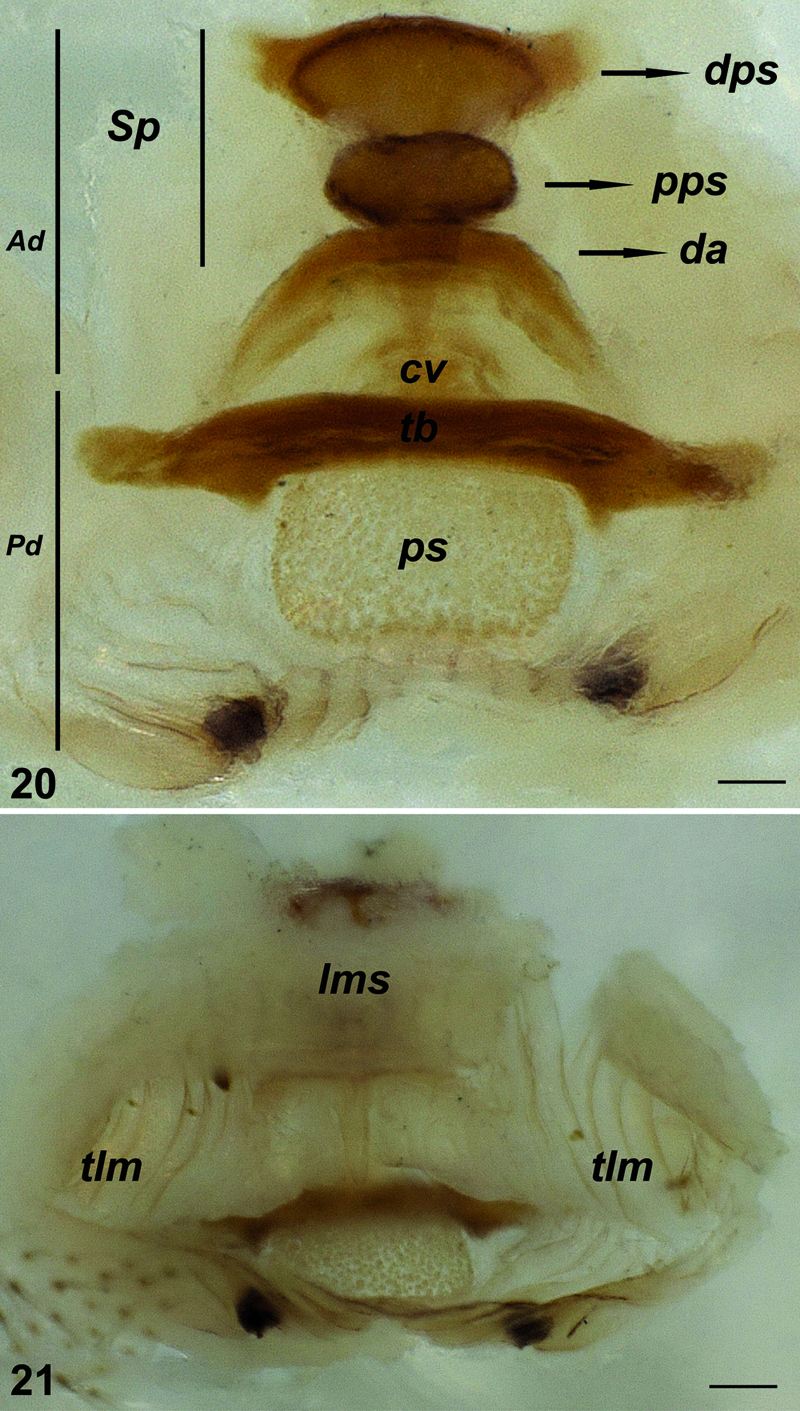
Vulva of Hygrocrates deelemanus sp. n. **20** dorsal view **21** ventral view. Abbreviations: Ad anterior diverticulum Pd posterior diverticulum Sp spermatheca dps distalmost part of spermatheca pps proximalmost part of spermatheca da dorsal arch cv central valve tb transverse bar ps membranous sac lms large membranous sac tlm twisted lateral membranes. Scale lines: 0.1 mm.

Palpal organ with pyriform bulbus and hook-shaped (tapering towards the tip) embolus. Bulbal apophyses are more strongly sclerotized than the embolus (Figs 16–19). Vulva with two parts: anterior diverticulum and posterior diverticulum (Fig. 20). Anterior diverticulum consists of a dorsal arch with spermathecae which have two parts (distalmost and proximalmost parts), a large membranous sac (clearly visible in dorsal view) and widened twisted lateral membranes (Fig. 21). Posterior diverticulum consists of a central valve with a transverse bar, a wide membranous sac and a couple of small lateral membranous pockets (Fig. 20).

**Table 1.** Leg measurements of Hygrocrates deelemanus sp. n.

(Holotype ♂ / Paratype ♀)	Fe	Pa	Ti	Me	Ta
Leg I	2.32 / 2.52	1.60 / 1.66	2.00 / 2.43	2.24 / 2.30	0.64 / 0.63
Leg II	2.08 / 2.32	1.48 / 1.44	1.80 / 1.84	2.08 / 1.96	0.56 / 0.60
Leg III	1.76 / 1.92	0.88 / 1.12	1.20 / 1.40	1.60 / 1.88	0.56 / 0.60
Leg IV	2.40 / 2.48	1.36 / 1.36	1.88 / 2.08	2.20 / 2.60	0.59 / 0.61

### Hygrocrates lycaoniae

(Brignoli, 1978)

**Figures 22–29. F8:**
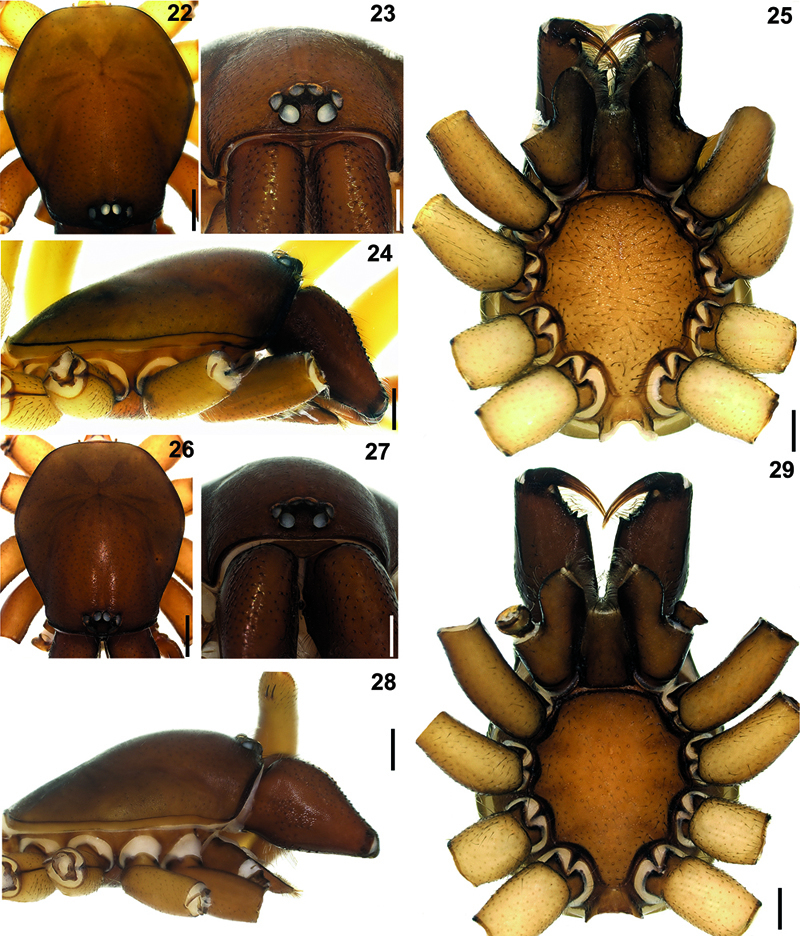
Hygrocrates lycaoniae **22** (♂), **26** (♀) carapace, dorsal view **23** (♂), **27** (♀) ditto, anterior view **24** (♂), **28** (♀) ditto, lateral view **25** (♂)**, 29** (♀) ditto, ventral view. Scale lines: (22, 24, 25, 26, 28, 29) 0.5 mm; (23, 27) 0.25 mm

Figs 22 [Fig F8] [Fig F9] 34 36, 38

Harpactocrates lycaoniae : Brignoli, 1978: 463, f. 2, 8 (D ♂).
Harpactocrates lycaoniae .: Deeleman-Reinhold & Deeleman, 1988: 240, f. 13, 22, 320–325 (T ♂ from *Harpactocrates*, D ♀).
Harpactocrates lycaoniae .: Dunin, 1992: 41, f. 3 (♂ ♀).

#### Material Examined:

1 ♂, 1 ♀ (SMF) (abdomen heavily damaged during dissection) **Burdur Province**, Yeşilova District, side of Salda Lake [37°30'32.78"N, 29°41'56.66"E], 14.VII.2010, leg. E.A.Yağmur & M. Elverici.

#### Diagnosis:

Hygrocrates lycaoniae can be distinguished from Hygrocrates caucasicus by the pyriform shape of the bulbus (bulbus smooth and cylindrical in Hygrocrates caucasicus) (see Dunin 1992); and from Hygrocrates georgicus by having apically oval-shaped spermathecae (see Mcheidze 1972).

#### Note:

Hygrocrates caucasicus was originally described on the basis of two males by Dunin (1992). The females of this species have not been collected yet, but the bulbal structures of the male palp were well illustrated by Dunin (1992). However, the information of Hygrocrates georgicus is still insufficient. The following information was given by Dunin (1992: p. 60) in his review of the Caucasian Dysderidae (our translation!): *“H. georgicus was described on the basis of the female holotype from Georgia: the vicinity of Tbilisi, Kodzhori, Udzho. The holotype was lost. The species was tentatively placed in Hygrocates by Deeleman-Reinhold and Deeleman (1988). To confirm their placement addition material is required. This species is absent from my collection.”* During the preparation of this paper, as a result of our correspondence with the Tbilisi Janashia Museum (Georgia) which retains the spider collection of Tamara Mcheidze, it is obvious that the holotype of this species is lost (S. Otto pers. comm.). Thus, we could not examine it, but on the basis of the original illustrations of the vulva by Mcheidze (1972), Hygrocrates georgicus can be distinguished from the Turkish members of the genus by the linear distalmost part of spermathecae.

#### Measurements (♂ / ♀):

AL 3.36 / ?; CL 2.40 / 3.00; CWmax 2.20 / 2.44; CWmin 1.52 / 1.76; AMEd 0.14 / 0.16; PLEd 0.13 / 0.15; PMEd 0.11 / 0.12; ChF 0.77 / 0.79; ChG 0.45 / 0.50; ChL 1.30 / 1.46. Leg measurements are given in Table 3.

#### Description:

General features of the body of Hygrocrates lycaoniae closely resemble the new species (Figs 22–29),Hygrocrates deelemanus sp. n., but the two are easily differentiated by their different body sizes and by structures of the male and female genitalia (Figs 30–33, 35–38). The males of the two species are easily distinguished in ventral view (90° angle) by the terminal part of the bulbus having the following characteristics:

1. Embolic base is pear-shaped in the two species, but the tip of the embolic base located at 12 o’clock in Hygrocrates deelemanus sp. n. (Fig. 37) and at 10 o’clock in Hygrocrates lycaoniae (Fig. 38).

2. Apophysisa and Apophysisb are short and blunt in Hygrocrates deelemanus sp. n. (Fig. 37), but long in Hygrocrates lycaoniae (Fig. 38).

3. Apophysisb originates near the tip of the embolic base in Hygrocrates deelemanus sp. n. (Fig. 37), but originates from the central part of the tip of the embolic base in Hygrocrates lycaoniae (Fig. 38).

4.Embolus is curved between Apophysisa and Apophysisb in Hygrocrates deelemanus sp. n. (Fig. 37), but is raised from the embolic base and separated from Apophysisa and Apophysisb in Hygrocrates lycaoniae (Fig. 38).

The females of the two species are easily distinguished by the form of the proximalmost part of the spermathecae which is oval in Hygrocrates deelemanus sp. n. (Figs 20–21) and circular in Hygrocrates lycaoniae (Fig. 34). Details of leg spination are given in Table 4.

**Figures 30–33. F9:**
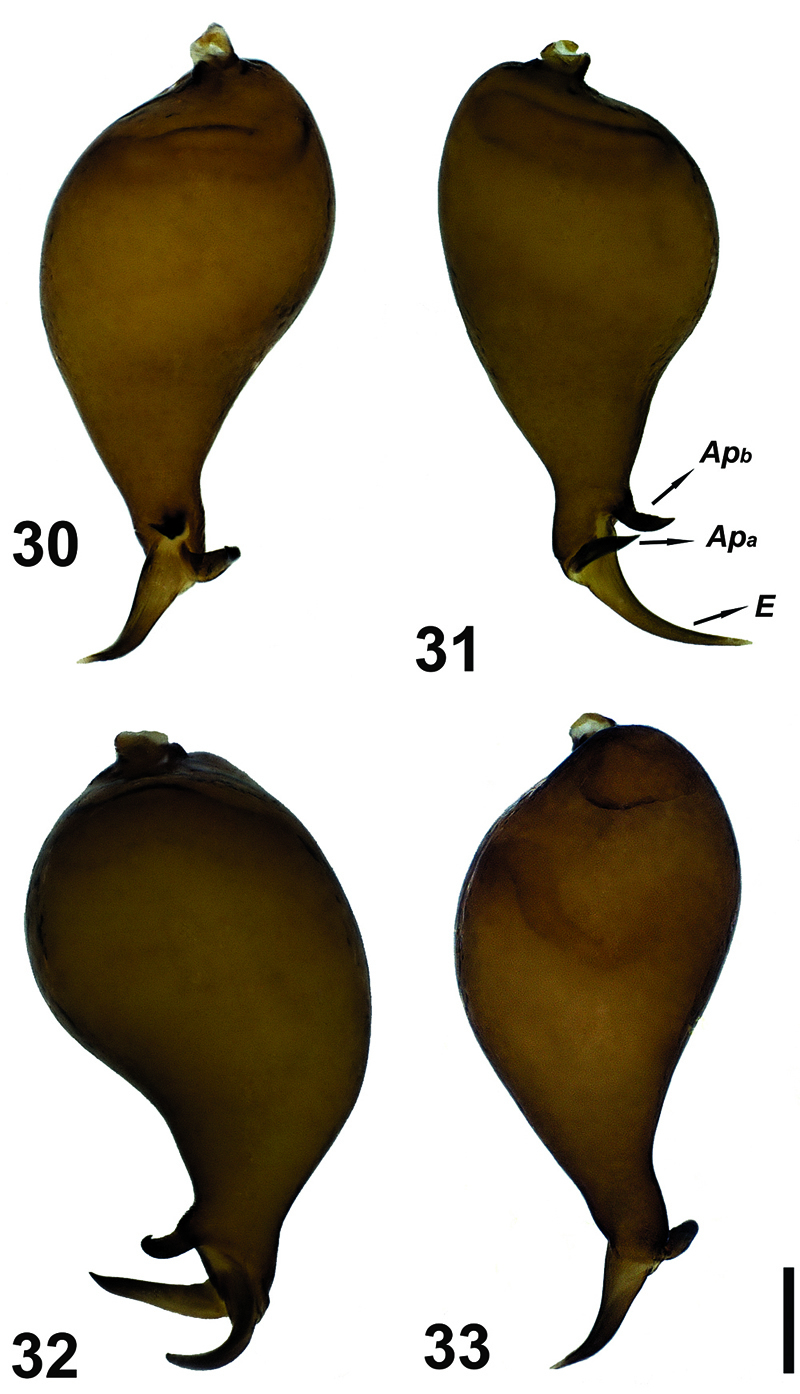
Male palp of Hygrocrates lycaoniae. Abbreviations: Apa apophysisa Apb apophysisb E embolus. Scale line: 0.1 mm.

**Figure 34.**
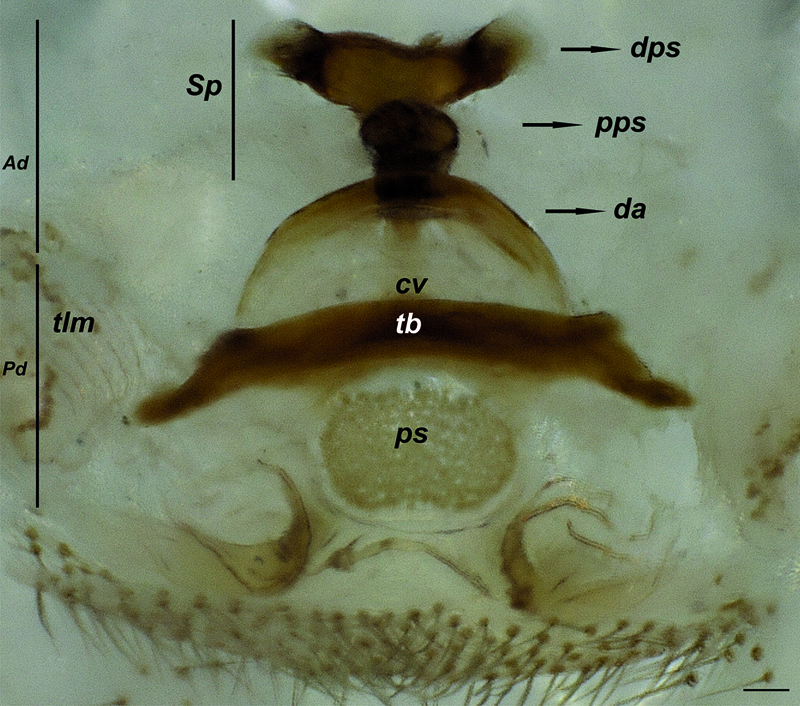
Vulva of Hygrocrates lycaoniae, dorsal view. Abbreviations: Ad anterior diverticulum Pd posterior diverticulum Sp spermatheca dps distalmost part of spermatheca pps proximalmost part of spermatheca da dorsal arch cv central valve tb transverse bar ps membranous sac tlm twisted lateral membranes. Scale line: 0.1 mm

**Table 2.** Leg spination of Hygrocrates deelemanus sp. n.

♂ (Holotype)	Leg I	Leg II	Leg III	Leg IV
C	0	0	0	0
Tr	0	0	0	0
Fe	Pl 1	Pl 0–1	D 1	D 1, 1
Pa	0	0	0	0
Ti	0	0	Pl 1 Rl 1, 2 V 1, 2	Pl 2, 2 Rl 2, 1 V 1, 1
Me	0	0	Pl 1, 1 D 2, 2 Rl 1, 2, 1, 1 V 2, 2	Pl 5 Rl 5 V 2
Ta	0	0	0	0
♀ (Paratype)				
C	0	0	0	0
Tr	0	0	0	0
Fe	Pl 2	Pl 1	0	D 1, 1
Pa	0	0	0	0
Ti	0	0	Pl 1 Rl 1, 2 V 1, 2	Pl 2, 2 Rl 1, 1 V 1, 2
Me	0	0	Pl 1, 1 Rl 1, 1, 1, 1 V 1, 2	Pl 5 Rl 5 V2
Ta	0	0	0	0

**Table 3.** Leg measurements of Hygrocrates lycaoniae

(♂ / ♀)	Fe	Pa	Ti	Mt	Ta
Leg I	2.28 / 2.24	1.40 / 1.44	1.84 / 1.96	2.36 / 1.92	0.53 / 0.60
Leg II	2.08 / 2.00	1.44 / 1.40	1.85 / 1.72	1.92 / 1.80	0.50 / 0.56
Leg III	1.73 / 1.68	1.10 / 0.92	1.29 / 1.16	1.58 / 1.52	0.50 / 0.48
Leg IV	2.19 / 2.20	1.20 / 1.20	1.83 / 1.76	2.20 / 2.12	0.60 / 0.56

**Figures 35–38.**
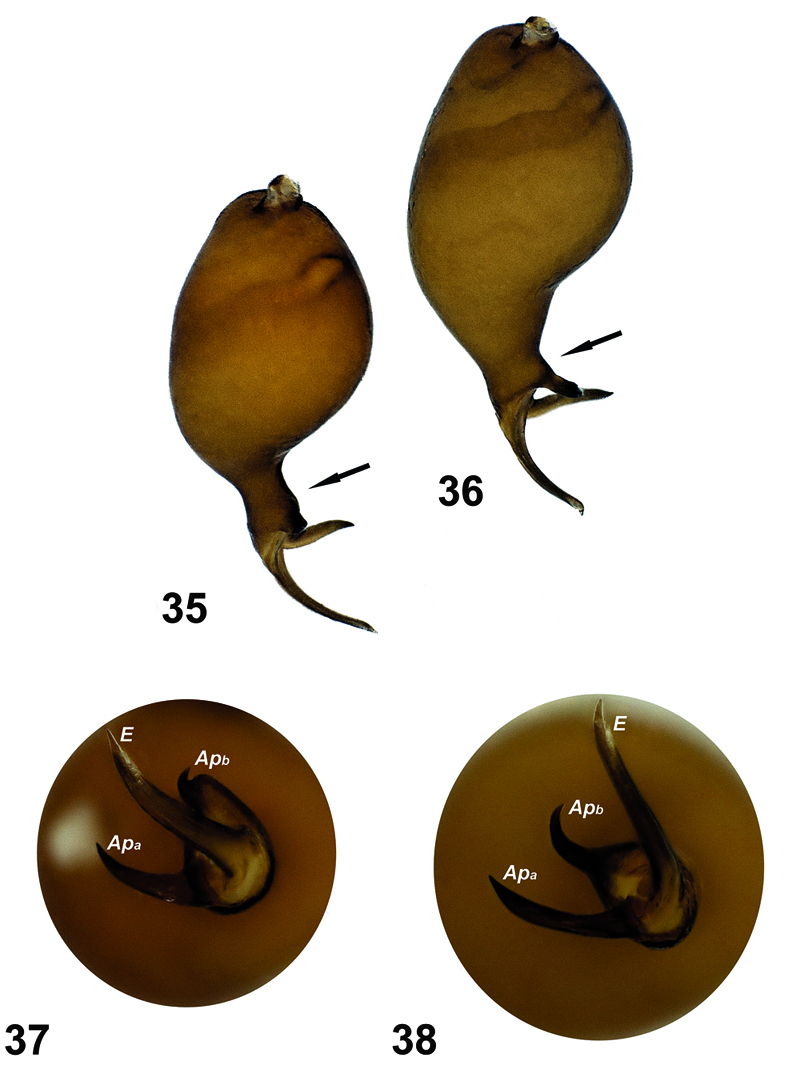
Comparison of the palps of the two species. **35, 37** Hygrocrates deelemanus sp. n. **36, 38** Hygrocrates lycaoniae. Abbreviations:Apa apophysisa Apb apophysisb E embolus.

## Habitat and distribution

**Figures 39–42.**
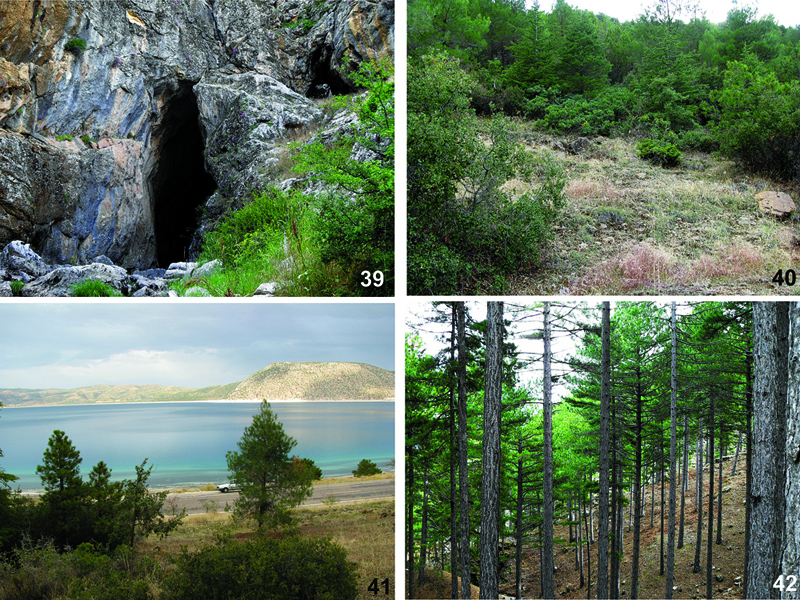
Habitats ofHygrocrates species. **39** The type locality of Hygrocrates lycaoniae in Konya province**40, 41** Localities of Hygrocrates lycaoniae in Burdur province **42** The type locality of Hygrocrates deelemanus sp. n.

The holotype of Hygrocrates lycaoniae was collected from outside of Körükini Cave, which is located in Çamlık Village (Beyşehir District, Konya Province). The vegetation surrounding the cave is the mixed forest composed of Taurus fir, juniper, oak and maple species. Annual and perennial herbaceous plants also grow very densely around the cave (Fig. 39). Although several field trips were conducted to the type locality, we failed to find any specimensof Hygrocrates lycaoniae. However, our male and female specimens of Hygrocrates lycaoniae were collected near to Salda Lake (Burdur Province), which is approximately 170 km apart from the type locality (Figs 40, 41). The specimens were collected in the leaf-litter of shrublands surrounding the Salda Lake. Deeleman-Reinhold and Deeleman (1988) collected their samples of Hygrocrates lycaoniae from leaf litter and under stones of wetland areas in Rhodes island. Therefore, it is safe to conclude that Hygrocrates lycaoniae prefers wetland habitats. We collected the samples of the new species, Hygrocrates deelemanus sp. n., from leaf litter under Pinus nigra trees (Fig. 42). Therefore it can be concluded that the two species are distributed in different habitat types of the Mediterranean.

**Table 4.** Leg spination of Hygrocrates lycaoniae

	Leg I	Leg II	Leg III	Leg IV
♂				
C	0	0	0	0
Tr	0	0	0	0
Fe	Pl 2	Pl 1	D 1	D 2
Pa	0	0	0	0
Ti	0	0	Pl 1 Rl 1, 2 V 1, 2	Pl 2, 2 Rl 1, 1, 1 V 1, 2
Me	0	0	Pl 1, 1, 1 Rl 1, 1, 1 V 2, 2	Pl 1, 1, 1, 1 Rl 5 V 2
Ta	0	0	0	0
♀				
C	0	0	0	0
Tr	0	0	0	0
Fe	Pl 1–2	Pl 1	0	D 2
Pa	0	0	0	0
Ti	0	0	Pl 1 Rl 1, 2 V 1, 2	Pl 2, 2 Rl 2, 1 V 1, 2
Me	0	0	Pl 1 Rl 1, 1, 1 V 2, 2	Pl 1, 1, 1 Rl 1, 1, 1, 1 V 2, 2
Ta	0	0	0	0

## Discussion

In general, the taxonomy of the subfamily Dysderinae is well defined (see Deeleman-Reinhold and Deeleman 1988; Dunin 1992). To date, 24 Dysderinae species, including the newly described species, have been recorded from Turkey in the following four genera: Dysdera Latreille, 1804 (20 species), Dysderocrates Deeleman-Reinhold & Deeleman, 1988 (1 species), Harpactocrates (1 species) and Hygrocrates (2 species) (Bayram et al. 2010; present data). The diversity of Dysderinae is not very high compared to neighbouring Greece (36 species: 34 Dysdera species, 2 Dysderocrates species) and Georgia (28 species: 3 Cryptoparachtes species, 21 Dysdera species, 1 Harpactocrates species and 2 Hygrocrates species). Nevertheless, the Turkish Dysderinae fauna includes more species than the other neighbouring countries, such as Azerbaijan (17 species), Bulgaria (14 species) and Armenia (4 species) (Otto and Dietzold 2006; Helsdingen 2010).

## Supplementary Material

XML Treatment for Hygrocrates deelemanus

XML Treatment for Hygrocrates lycaoniae
